# Optimization of Processing Parameters and Application Performance Evaluation of a High Thermal Conductivity, Low Thermal Resistance Gel

**DOI:** 10.3390/gels12040293

**Published:** 2026-03-31

**Authors:** Yuwen Xu, Danni Hong, Liangjun Liu, Wenfei Wang, Minghua Jiang, Haibing Yang, Tingxin Chen, Kun Jia

**Affiliations:** 1 School of Materials Science and Engineering, South China University of Technology, Guangzhou 510641, China; 202411085466@mail.scut.edu.cn; 2 China Science and Technology on Reliability Physics and Application of Electronic Component Laboratory, Guangzhou 511370, China; hongdanni@ceprei.com; 3 U-Bond Technology Inc., Dongguan 523800, China; liulj@ubondtech.com (L.L.); wangwf@ubondtech.com (W.W.); kevin.jiang@ubondtech.com (M.J.); harry.yang@ubondtech.com (H.Y.); 4 School of Materials Science and Engineering, University of Electronic Science and Technology of China, Chengdu 611731, China; jiakun@uestc.edu.cn

**Keywords:** thermal interface materials (TIMs), thermal conductive gel, interfacial thermal resistance, filler ratio, wetting time, silicone oil viscosity

## Abstract

Thermal interface materials (TIMs) are essential for addressing heat dissipation challenges in high-performance electronic devices. Among various TIMs, thermal conductive gels exhibit significant potential in high heat flux applications due to their excellent flexibility and superior gap-filling capability. Current research primarily concentrates on the fabrication and performance characterization of novel thermal conductive gels, while comparatively little attention has been devoted to the optimization of processing parameters. Furthermore, existing characterization methods often fail to accurately replicate real-world operating conditions, resulting in discrepancies between laboratory measurements and actual performance. An orthogonal experimental design was adopted to systematically elucidate the influence of filler ratio, wetting time, and silicone oil viscosity on the bonding strength of thermal conductive gels. The filler ratio exerts the most significant influence, followed by silicone oil viscosity and wetting time. Subsequently, the thermal conductivity and thermal resistance of both commercial thermal conductive gels and the as-prepared gels were characterized using the steady-state heat flow method and the double-interface method, respectively. Under the optimized preparation conditions (filler ratio of 88%, silicone oil viscosity of 600 cP, and wetting time of 14 h), the self-developed thermal conductive gel exhibits a thermal conductivity of 3.75 W·m^−1^·K^−1^ and a bonding strength of 0.248 MPa, outperforming commercial counterparts and demonstrating promising application potential. It was further concluded, through comparisons of curing rheology and long-term reliability evolution with commercial counterparts, that the self-developed thermal conductive gel possesses enhanced stability and reliability. This study provides a practical reference for the development and engineering application of high thermal conductivity, low thermal resistance gels.

## 1. Introduction

With the continuous advancement of electronic components toward higher power density, miniaturization, and greater integration, heat flux density has increased significantly, rendering thermal management of chips an increasingly critical challenge [1,2,3]. In practical applications, the actual contact area between a chip and a heat sink accounts for only approximately 10% of the apparent macroscopic contact area, while the remaining interfacial region is filled with air, which exhibits an extremely low thermal conductivity of only 0.03 W·m^−1^·K^−1^ [4,5,6]. Such interfacial thermal resistance severely impedes heat dissipation efficiency. Previous studies have reported that the reliability of an electronic system may decrease by nearly 50% for every 10 °C increase in operating temperature [7,8]. Therefore, the development of high-performance thermal interface materials (TIMs) for high-power chips has become a prominent research focus. TIMs primarily include thermal conductive pads, thermal conductive silicone grease, and thermal conductive gels. Among these, thermal conductive silicone grease often suffers from structural instability during long-term operation due to the oil-pumping effect [9]. In contrast, the relatively large thickness and high elastic modulus of thermal conductive pads tend to increase interfacial contact thermal resistance [10,11]. Compared with thermal conductive pads and silicone grease, thermal conductive gels exhibit unique advantages in high heat flux applications owing to their intrinsic flexibility, high thermal conductivity potential, and excellent gap-filling capability. Consequently, they demonstrate irreplaceable application value in 5G communication base stations, artificial intelligence servers, and power modules for new energy vehicles, among other high-heat-flux-density scenarios [12,13,14].

**Figure 1:**
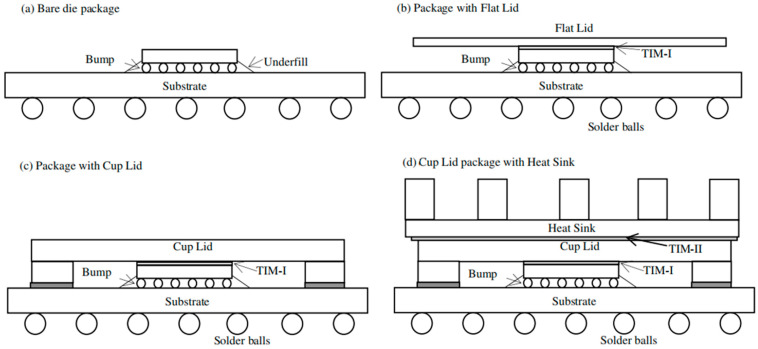
Various package lids and TIM-I in electronic packages modeled [27] @2008 Elsevier Ltd. (Amsterdam, The Netherlands) All rights reserved.

At present, research on thermal conductive gels primarily focuses on enhancing their thermal conductivity. The main strategies include incorporating nano- or micro-scale thermal conductive fillers, such as boron nitride, aluminum oxide, and silicon carbide, as well as developing novel polymer matrices (e.g., modified epoxy resins and silicone-based systems) to optimize the mechanical properties and thermal stability of the materials [15,16,17,18,19,20,21]. For thermal conductive gels, practical applications require consideration not only of their intrinsic properties but also of their process compatibility. It has been widely reported that hybrid particle size distributions play a critical role in governing both thermal transport and rheological performance. Specifically, larger fillers establish continuous heat-conduction pathways, whereas smaller particles fill the interstitial spaces, leading to a denser packing structure with reduced void content. Such a hierarchical packing configuration not only facilitates the formation of efficient thermal conduction networks but also improves flowability and extrudability. As a result, thermally conductive gels prepared via this strategy exhibit simultaneously enhanced thermal conductivity and desirable rheological characteristics [22,23,24]. Beyond the design of particle size distribution, processing strategies are equally critical in determining the final performance of thermally conductive gels. In a typical procedure, fillers are first surface-treated to improve interfacial compatibility and then gradually incorporated into the silicone matrix through prolonged mixing (≈120 min). The mixture is subsequently heated above 100 °C to eliminate residual moisture, followed by the addition of other functional additives. Such well-controlled filler treatment and dispersion processes are vital for ensuring homogeneous microstructures, which in turn significantly influence both thermal conductivity and rheological behavior [25,26]. As shown in Figure 1, the selection of thermal interface materials employed between the die and the lid (commonly referred to as TIM-I) involves multiple critical factors. In addition to filler engineering and processing strategies, particular attention must be paid to the rheological behavior of the thermally conductive gel and the filler loading ratio, both of which play a decisive role in determining processability and thermal performance [27,28]. It is primarily influenced by processing parameters, including filler ratio, silicone oil viscosity, and wetting time [29]. The bonding strength reflects the interfacial reliability between the thermal conductive gel and the chip and can serve as a key mechanical parameter for evaluating processing adaptability [30,31,32].

However, the underlying mechanisms and governing principles by which processing parameters influence the viscosity of thermal conductive gels remain insufficiently understood. In addition, current application evaluations predominantly focus on the intrinsic material properties, which makes it difficult to accurately simulate the influence of thermal conductive gels on device junction temperature and thermal resistance under realistic operating conditions. Consequently, discrepancies often arise between laboratory evaluation results and actual service performance. Therefore, to achieve high-performance thermal conductive gels, it is essential to systematically investigate both process optimization strategies and more application-oriented evaluation methods.

In light of the application requirements of TIM-I, this study systematically investigates three critical parameters governing the performance of thermally conductive gels, including silicone oil viscosity, filler ratio, and processing-induced wetting time. These factors are closely associated with the thermal conductivity, rheological properties, and interfacial adhesion strength of the material. An orthogonal experimental design was adopted to optimize the formulation and processing conditions. Under the optimized conditions (88% filler loading, silicone oil viscosity of 600 cP, and a wetting time of 14 h), the as-prepared thermally conductive gel achieves a thermal conductivity of 3.75 W·m^−1^·K^−1^ along with a bonding strength of 0.248 MPa, demonstrating a favorable balance between thermal and mechanical performance.

Subsequently, the thermal conductivity and thermal resistance of both commercial thermal conductive gels and the as-prepared gels were characterized using the steady-state heat flow method and the double-interface method, respectively. A commercial thermally conductive gel was selected as a benchmark for comparative analysis, including curing rheology and processing conditions, intrinsic thermal conductivity, and operational thermal resistance. In addition, reliability assessments were performed under stringent chip-level conditions, namely thermal cycling tests (TCT, 1000 cycles), high-temperature storage tests (HTST, 1000 h), and unbiased highly accelerated stress tests (uHAST, 384 h). The developed gel exhibits performance comparable to that of the commercial counterpart, particularly in terms of thermal conductivity, elongation at break, and adhesion strength, indicating its strong potential for practical TIM-I applications.

## 2. Results and Discussion

### 2.1. Process Optimization and Preparation of Thermal Conductive Gel

As presented in Table 1, to systematically evaluate the influence of filler loading, silicone oil viscosity, and wetting time on the performance of thermal conductive gels, a three-factor, three-level orthogonal experimental design was employed, with these processing parameters treated as independent variables [33,34,35]. Nine formulations were thus prepared, and their rheological behavior (viscosity), adhesion strength, and thermal conductivity were comprehensively characterized, with the results duly recorded.

**Figure 2:**
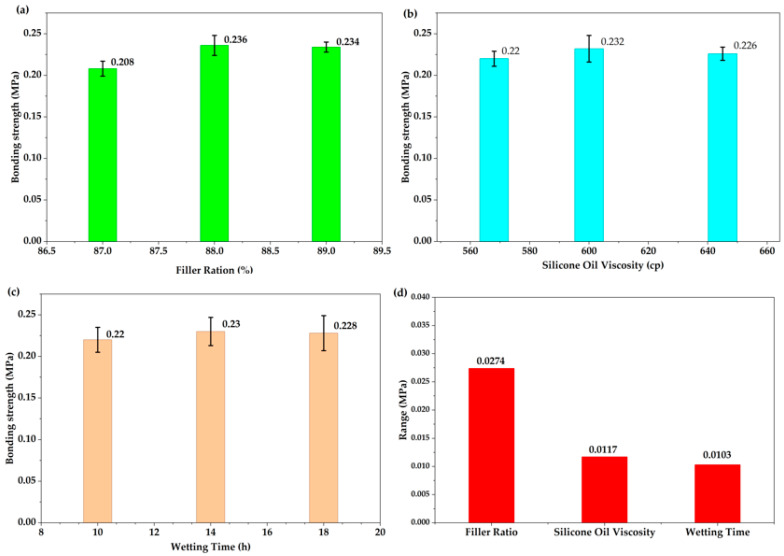
Influence trends and ranges of different factors on the shear strength of thermal conductive gels: (**a**) Filler ratio, (**b**) silicone oil viscosity, (**c**) wetting time, and (**d**) ranges of the three factors.

Range analysis was conducted to evaluate the influence of filler ratio, silicone oil viscosity, and wetting time on the bonding strength of the thermal conductive gel. The range value reflects the degree of variation in the experimental index; a larger range indicates a more significant effect of the corresponding factor on bonding strength. Therefore, the relative importance of the three factors can be ranked according to their range values. The results of the range analysis are presented in Figure 2d. The calculated ranges for filler ratio, silicone oil viscosity, and wetting time were 0.0274 MPa, 0.0117 MPa, and 0.0103 MPa, respectively. These results indicate that the filler ratio is the most influential factor affecting bonding strength, followed by silicone oil viscosity, while wetting time exhibits the least effect. As shown in Figure 2a, the bonding strength of the thermal conductive gel initially increases and subsequently decreases with increasing filler ratio. This phenomenon is primarily attributed to the bonding strength of the thermal interface material, which fundamentally depends on the interfacial interactions between the filler particles and the silicone oil matrix. A higher density of contact sites between the filler and the silicone oil enhances mechanical interlocking and interfacial adhesion, thereby improving the overall bonding strength. However, when the filler content is further increased beyond an optimal level, particle agglomeration may occur, leading to non-uniform dispersion and a consequent reduction in effective interfacial adhesion.

As shown in Figure 2b, silicone oil viscosity governs the degree of molecular chain entanglement and fluidity within the matrix. An increase in viscosity generally corresponds to longer molecular chains, which enhances adsorption and interfacial entanglement with the filler surface, thereby strengthening interfacial cohesion. Nevertheless, excessively high viscosity compromises flowability, hindering the complete wetting of filler particles. This insufficient wetting reduces the effective interfacial contact area between the matrix and filler, ultimately weakening the bonding strength. Compared with the filler, the silicone oil matrix cannot substitute for the mechanical framework established by the filler network. Since the structural integrity and load-bearing capability are predominantly determined by the filler skeleton, the influence of silicone oil viscosity is inherently constrained, resulting in a weaker overall effect than that of the filler ratio.

As shown in Figure 2c, wetting time exhibits the least influence among the three factors. Its role is primarily associated with the infiltration and wetting process of silicone oil onto the filler surface, which is characterized by a saturation effect. Insufficient wetting (e.g., within 10 h) results in incomplete interfacial infiltration, leading to the formation of interfacial voids and consequently reduced bonding strength. When the wetting time is extended to approximately 14 h, the silicone oil sufficiently envelops the filler particles and effectively fills the interfacial gaps, thereby enhancing interfacial integrity. Further prolonging the wetting time to 18 h does not significantly improve the infiltration behavior or interfacial bonding quality, indicating that the wetting process has reached equilibrium. Based on the range analysis results, the optimal preparation parameters were determined to be a filler ratio of 88 wt%, a silicone oil viscosity of 600 cP, and a wetting time of 14 h. Under these conditions, the bonding strength reached 0.248 MPa.

**Figure 3:**
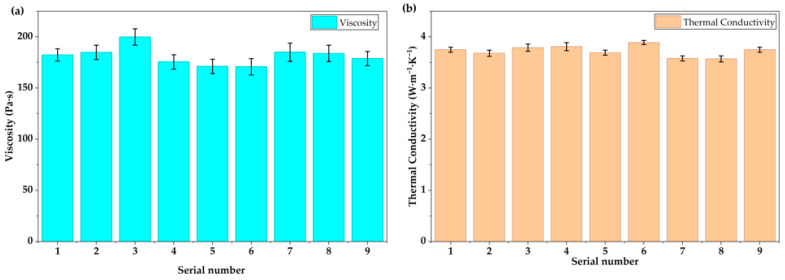
Viscosity and thermal conductivity data of 9 groups of gels in orthogonal experiments: (**a**) the viscosity of the gels; (**b**) the thermal conductivity of the gels.

As shown in Figure 3a, the gel in serial no. 3 exhibits the highest viscosity, followed by serial nos. 2 and 7, indicating that the viscosity of the gel is not only related to the wetting time but also strongly dependent on the silicone oil viscosity. From Figure 3b, the gel in Run 6 delivers the maximum thermal conductivity, with serial nos. 3 and 4 ranking next, revealing that thermal conductivity is primarily governed by the filler loading. Subsequently, range analysis was performed on the three performance indicators (bonding strength, viscosity, and thermal conductivity) across the 9 orthogonal experimental serial numbers. The calculated range values were 11.16 Pa·s for viscosity, 0.0634 W·m^−1^·K^−1^ for thermal conductivity, and 0.022 MPa for bonding strength. Based on these results, serial no. 1 was determined to be the optimal condition, where the prepared gel achieved a viscosity of 182.4 Pa·s, a bonding strength of 0.248 MPa, and a thermal conductivity of 3.75 W·m^−1^·K^−1^. For the subsequent performance comparison, Gel A was prepared under serial no. 1 conditions.

### 2.2. Rheological Properties

**Figure 4:**
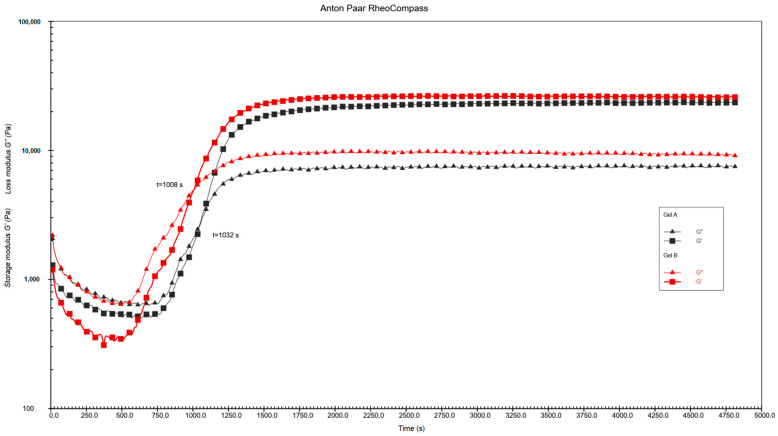
Rheological Curing Curves of Gel A and Gel B.

The time-sweep rheological measurements were conducted to investigate the sol–gel transition behavior of Gel A and Gel B, as illustrated in Figure 4. For both samples, the storage modulus (G′) and loss modulus (G″) exhibited a characteristic evolution during the gelation process: initially, G″ > G′, indicating a dominant liquid-like (sol) state with viscous behavior. As the reaction proceeded, both G′ and G″ increased sharply, and the crossover point (G′ = G″) was reached, which is defined as the gel point. Specifically, Gel B showed a shorter gelation time of 1008 s, while Gel A exhibited a slightly delayed gel point at 1032 s, demonstrating that the formulation of Gel B accelerates the sol–gel transition. After the gel point, both G′ and G″ reached a stable plateau, with G′ consistently exceeding G″ by approximately one order of magnitude, confirming the formation of a robust elastic gel network with solid-like viscoelastic properties. Notably, Gel B achieved a higher equilibrium G′ value (~3 × 10^4^ Pa) than Gel A (~2.5 × 10^4^ Pa), along with a higher equilibrium G″, indicating a stronger crosslinked network structure and enhanced mechanical rigidity of the Gel B system.

### 2.3. Characterization of Intrinsic Thermal Conductivity of Thermal Conductive Gels

**Figure 5:**
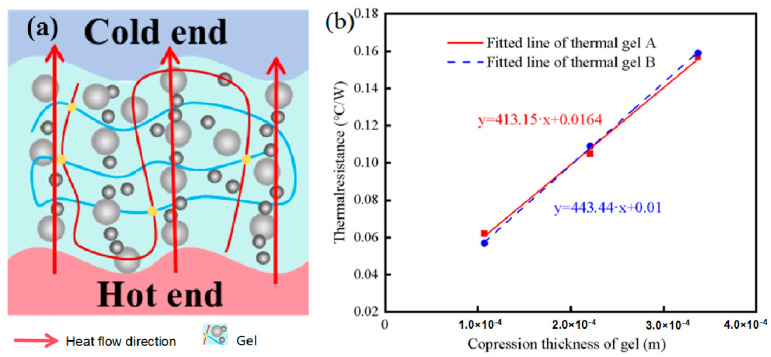
Schematic diagram and test results of the steady-state heat flow measurement for thermal conductive gels: (**a**) Schematic diagram of steady-state heat flow measurement for thermal conductive gels, (**b**) Variation in gel thermal resistance with compressed thickness.

As shown in Figure 5a, the thermal conductivity of thermal conductive gel A and thermal conductive gel B was measured using a steady-state heat flow apparatus. During the test, the temperature of the hot side was maintained at 50 °C. The gap distance between the hot and cold sides was adjusted to 0.337 mm, 0.221 mm, and 0.107 mm, to test three samples for each group, respectively. Under steady-state conditions, the input power and the temperature at the cold side were recorded for thermal conductivity calculation.

According to Equation (1), the thermal resistance of the two thermal conductive gels at different compression thicknesses was calculated, and the corresponding results are summarized in Table 2.

(1) $$R=\frac{T_{2}-T_{1}}{P}$$

In Equation (1), *R* denotes the thermal resistance, *T*_1_ and *T*_2_ represent the temperatures of the hot and cold sides, respectively, and *P* corresponds to the input power applied to the thermal conductive gel.

As shown in Figure 5b, the thermal resistance of both thermal conductive gels increases linearly with increasing compression thickness. In the steady-state heat flow measurement, the total thermal resistance of the thermal conductive gel is primarily composed of intrinsic (bulk) thermal resistance and interfacial thermal resistance, as described by Equations (2)–(4) [36,37].

(2) $$R=R_{1}+R_{0}$$

(3) $$R_{1}=d/(\lambda \cdot A)$$

(4) $$R=d/\lambda +R_{0}$$

In these equations, *R*_0_ and *R*_1_ represent the interfacial thermal resistance and intrinsic (bulk) thermal resistance, respectively; *d* denotes the sample thickness; λ is the thermal conductivity; and *A* corresponds to the heat dissipation area. By linearly fitting the thermal resistance values of thermal conductive gels A and B at different thicknesses, the relationships between thermal resistance and thickness were obtained, as expressed in Equations (5) and (6).

(5) y=413.15·x+0.0164

(6) y=444.54·x+0.0098

From the fitted equations, it can be inferred that the slopes of the two linear plots in Figure 5b are positively correlated with the reciprocal of the thermal conductivity of the respective thermal conductive gels. Based on the linear fitting results, the thermal conductivities of thermal conductive gels A and B were calculated to be 3.75 W·m^−1^·K^−1^ and 3.49 W·m^−1^·K^−1^, respectively.

**Figure 6:**
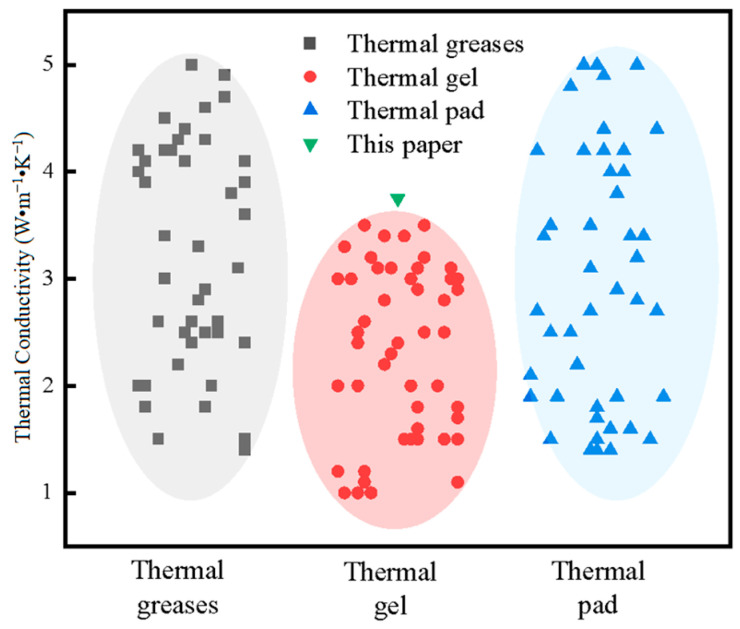
Comparison of thermal conductivity of different types of thermal interface materials.

As shown in Figure 6, previous studies by Tong, Yang, and other researchers have reported that the thermal conductivity of most existing thermal conductive gels is primarily distributed within the range of 1–3.5 W·m^−1^·K^−1^ [38,39]. Therefore, the self-developed thermal conductive gel exhibits a thermal conductivity approximately 7.1% higher than that of the commercial counterpart, demonstrating its superior heat transfer capability.

### 2.4. Thermal Resistance Test of Thermal Conductive Gel in Service

**Figure 7:**
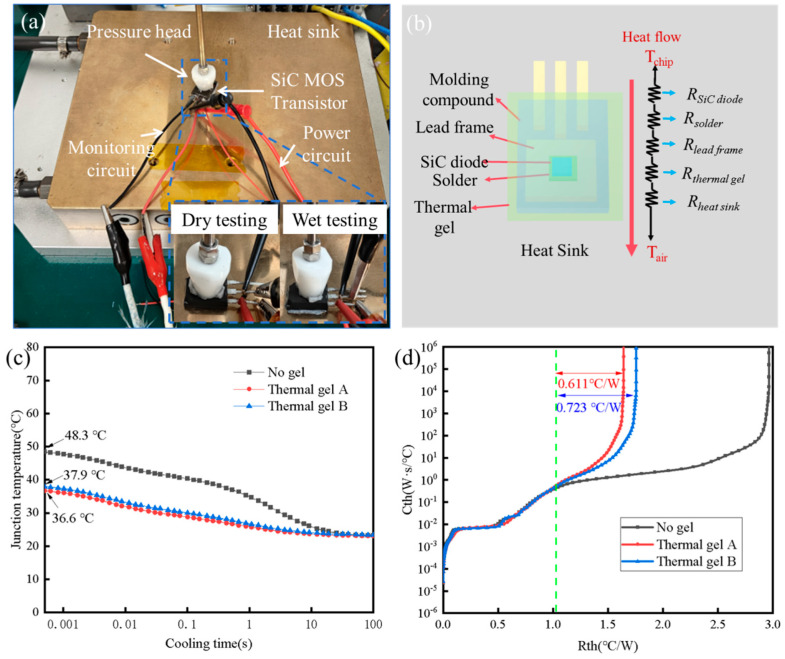
Dual-interface method test setup, thermal resistance network model, and test results for thermal conductive gels: (**a**) Dual-interface method test setup, (**b**) thermal resistance network model of the SiC diode, (**c**) junction temperature of the SiC diode, (**d**) in-service thermal resistance of the thermal conductive gels.

As shown in Figure 7, the service thermal resistance of the thermal conductive gels was evaluated using the double-interface method in accordance with the IEC 61189-2-208 standard [40]. A SiC diode with a constant power dissipation of 9 W and a bottom heat dissipation area of 1 cm^2^ was employed as the heat source. The junction temperature and thermal resistance of the device were recorded sequentially under dry and wet steady-state conditions. As illustrated in Figure 7c, the junction temperature of the SiC device under dry test conditions was 48.3 °C. After applying thermal conductive gel A and thermal conductive gel B (wet condition), the junction temperatures decreased to 36.6 °C and 37.9 °C, respectively.

For the SiC diode, natural convection from the top surface was considered negligible, and heat dissipation was assumed to occur sequentially through the chip, solder layer, lead frame, thermal conductive gel, and heat sink [41]. As illustrated in Figure 7b, the actual packaging structure of the SiC diode was modeled using a Cauer thermal network model. In this model, thermal capacitance represents the heat storage capability of each material layer. However, under steady-state heat transfer conditions, the effect of thermal capacitance can be neglected because the heat flow becomes time-independent. Therefore, in the steady state, the Cauer thermal network of the SiC diode simplifies to a series cascade of thermal resistances corresponding to the individual packaging layers [42,43]. Due to the differences in thermal conductivity and thermal capacitance among the constituent materials, distinct thermal resistance–thermal capacitance (R-C) characteristic curves are obtained. Based on the double-interface method and the corresponding transient thermal response curves, the thermal resistance of the thermal conductive gel can be extracted. As shown in Figure 7d, the service thermal resistances of thermal conductive gel A and thermal conductive gel B were determined to be 0.611 °C·W^−1^ and 0.723 °C·W^−1^, respectively. These results are consistent with the junction temperature analysis of the SiC diode discussed above. The lower thermal resistance of thermal conductive gel A indicates its superior heat dissipation capability compared with thermal conductive gel B.

### 2.5. Analysis of Heat Transfer Mechanism of Thermal Conductive Gel

**Figure 8:**
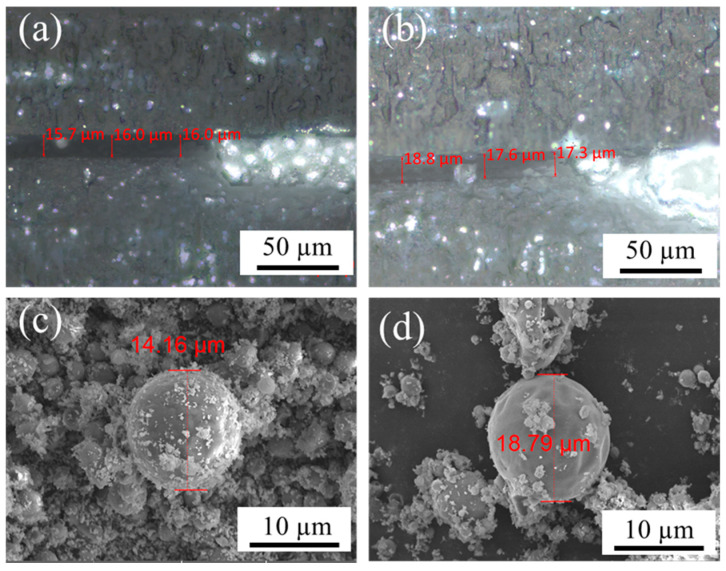
SEM images of the thermal conductive gels A (**a**,**c**) and B (**b**,**d**) the diameters of Al particles in the thermal conductive gels A and B.

To elucidate the origin of the difference in heat dissipation performance between thermal conductive gel A and thermal conductive gel B, the minimum bond-line thickness (BLT) of the two gels was characterized using an ultra-depth-of-field microscope. The corresponding results are presented in Figure 8a,b. The BLT values of thermal conductive gel A and thermal conductive gel B were measured to be 15.9 µm and 17.9 µm, respectively. According to Equation (3), the intrinsic (bulk) thermal resistances of thermal conductive gel A and thermal conductive gel B were calculated to be 0.042 °C·W^−1^ and 0.051 °C·W^−1^, respectively. According to Equation (4), the interfacial thermal resistances of thermal conductive gel A and thermal conductive gel B were calculated to be 0.569 °C·W^−1^ and 0.672 °C·W^−1^, respectively. In other words, thermal conductive gel A exhibits lower intrinsic (bulk) thermal resistance as well as lower interfacial thermal resistance compared with thermal conductive gel B.

To further elucidate the origin of the difference in heat dissipation performance between the two thermal conductive gels, the microstructural morphology of the fillers was analyzed after removing the organic matrix via thermal decomposition. As shown in Figure 8a,b, the thermal conductive fillers in both gels consist of large-sized Al particles and small-sized ZnO particles, forming a bimodal particle size distribution. Compared with thermal conductive gel B, the Al particles in thermal conductive gel A exhibit a smaller average diameter (14.16 µm) and are more uniformly coated by ZnO particles, resulting in a more homogeneous filler distribution. Considering that the thermal conductivity of vinyl silicone oil is only 0.2 W·m^−1^·K^−1^, whereas those of Al and ZnO are 247 W·m^−1^·K^−1^ and 5 W·m^−1^·K^−1^, respectively [44], heat conduction within the thermal conductive gels is predominantly governed by the thermally conductive filler network rather than the polymer matrix. As shown in Figure 8c,d, the reduced particle size of Al shortens the effective heat transfer distance and facilitates the formation of more continuous thermal conduction pathways. Consequently, the minimum BLT of the thermal conductive gel can be further reduced, which contributes to the overall decrease in intrinsic thermal resistance.

Moreover, the reduced particle size of Al increases the effective contact area between the thermal conductive gel and both the device surface and the heat sink. This enlarged interfacial contact area enhances phonon and electron coupling across the interface, thereby optimizing the heat dissipation pathway and effectively reducing the interfacial thermal resistance of the thermal conductive gel. In addition, the uniform dispersion of fine ZnO particles around the Al particles enables efficient filling of the interstitial voids between adjacent Al particles. This structural arrangement increases the packing density of the filler network and promotes the formation of more continuous thermal conduction pathways. As a result, the intrinsic (bulk) thermal resistance of the thermal conductive gel is further reduced.

### 2.6. The Long-Term Reliability Evaluation of Thermal Conductive Silicone Gel

**Figure 9:**
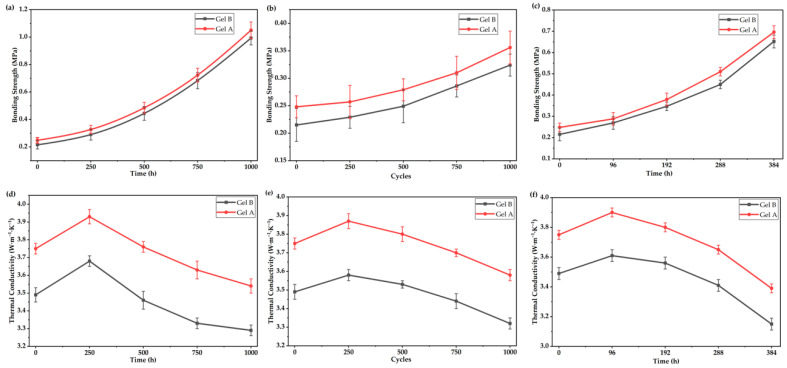
The long-term reliability evaluation of bonding strength was under (**a**) HTST, (**b**) TCT and (**c**) uHAST. And the evaluation of thermal conductivity was under (**d**) HTST, (**e**) TCT and (**f**) uHAST.

The long-term reliability of the bonding strength for Gel A and Gel B was systematically evaluated under three critical service conditions: HTST, TCT, and uHAST, as presented in Figure 9a–c, respectively. As shown in Figure 9a, both gels exhibited a continuous increase in bonding strength with prolonged storage time at high temperature, which can be attributed to the post-curing crosslinking reaction of the polymer matrix during long-term thermal exposure. Notably, Gel A consistently outperformed Gel B throughout the 1000 h storage period, achieving a final bonding strength of 1.05 MPa, which was approximately 5% higher than that of Gel B (0.993 MPa), demonstrating its superior long-term bonding stability under high-temperature conditions.

Figure 9b illustrates the evolution of bonding strength during thermal cycling tests. For both samples, the bonding strength increased gradually with the number of cycles, which is consistent with the further crosslinking of the gel network under alternating temperature stress. Gel A maintained a significantly higher bonding strength than Gel B across all test cycles, reaching a maximum value of 0.356 MPa after 1000 cycles, while Gel B only reached 0.324 MPa. The larger error bars observed for Gel B also indicate its poorer mechanical stability under thermal shock, further confirming the enhanced reliability of Gel A.

As depicted in Figure 9c, the uHAST test results revealed a similar trend: the bonding strength of both gels increased continuously with test time, and Gel A exhibited a consistently higher bonding strength than Gel B at all time points. After 384 h of uHAST testing, Gel A achieved a bonding strength of 0.696 MPa, which was ~7% higher than that of Gel B (0.652 MPa). The superior reliability of Gel A under all three reliability test conditions can be attributed to its optimized formulation (from orthogonal experiments), which promotes a more homogeneous and robust filler–polymer crosslinked network, effectively resisting the degradation caused by thermal aging, thermal stress, and hygrothermal environments.

Collectively, these results demonstrate that Gel A, prepared with the optimal process parameters, not only possesses excellent initial bonding performance but also exhibits outstanding long-term reliability under harsh service conditions, making it a highly promising adhesive material for practical applications.

Similarly, the thermal conductivity of the gels was measured following long-term reliability tests. As illustrated in Figure 9d, both gels exhibited a consistent trend of thermal conductivity evolution during HTST: an initial increase within the first 250 h, followed by a gradual decline over the remaining 750 h. This initial rise can be attributed to the post-curing crosslinking reaction of the polymer matrix, which enhances the interfacial bonding between fillers and the matrix, thereby optimizing the thermal conduction pathways. Notably, Gel A consistently maintained a significantly higher thermal conductivity than Gel B throughout the entire 1000 h test period, with a peak value of 3.93 W·m^−1^·K^−1^ at 250 h and a final value of 3.54 W·m^−1^·K^−1^, which was about 7.6% higher than that of Gel B (3.29 W·m^−1^·K^−1^) after 1000 h, demonstrating its superior thermal performance retention under long-term high-temperature exposure.

Figure 9e presents the thermal conductivity evolution during TCT. A similar trend was observed: both gels showed an initial increase in thermal conductivity up to 250 cycles, followed by a continuous decrease with further cycling. Gel A again outperformed Gel B at all test points, achieving a maximum thermal conductivity of 3.87 W·m^−1^·K^−1^ and retaining 3.58 W·m^−1^·K^−1^ after 1000 cycles, while Gel B only reached 3.58 W·m^−1^·K^−1^ at its peak and dropped to 3.32 W·m^−1^·K^−1^ after 1000 cycles. The larger error bars for Gel B also indicate poorer thermal stability under thermal shock, further confirming the enhanced reliability of Gel A.

As shown in Figure 9f, the uHAST test results revealed a comparable trend: the thermal conductivity of both gels increased initially (peaking at 96 h) and then decreased gradually with test time. Gel A maintained a consistently higher thermal conductivity than Gel B across all time points, reaching a peak of 3.9 W·m^−1^·K^−1^ and retaining 3.39 W·m^−1^·K^−1^ after 384 h of testing, which was about 8% higher than that of Gel B (3.15 W·m^−1^·K^−1^). The superior thermal reliability of Gel A under all three test conditions can be attributed to its optimized formulation derived from orthogonal experiments, which constructs a more homogeneous, robust, and interconnected filler–polymer crosslinked network. This structure effectively resists thermal aging, thermal stress-induced interface damage, and hygrothermal degradation, thereby preserving the continuous thermal conduction pathways and maintaining excellent thermal conductivity stability under harsh service conditions.

Overall, these results demonstrate that Gel A, prepared with the optimal process parameters, not only possesses excellent initial thermal conductivity but also exhibits outstanding long-term thermal reliability, making it a highly promising thermal interface material for practical applications in harsh environments.

## 3. Conclusions

In summary, the rheological and mechanical properties of the thermal conductive gel can be effectively regulated by adjusting the filler ratio, silicone oil viscosity, and wetting time. Among these parameters, the filler ratio exhibits the most significant influence on bonding strength, followed by silicone oil viscosity, while wetting time shows the weakest effect. Under the optimized preparation conditions (filler ratio of 88%, silicone oil viscosity of 600 cP, and wetting time of 14 h), the prepared gel achieved a viscosity of 182.4 Pa·s, a bonding strength of 0.248 MPa, and a thermal conductivity of 3.75 W·m^−1^·K^−1^.

The thermal conductivity and thermal resistance of the self-developed thermal conductive gel were systematically characterized using the steady-state heat flow method and the double-interface method, respectively. The thermal conductivity and overall thermal resistance were determined to be 3.75 W·m^−1^·K^−1^ and 0.611 °C·W^−1^, respectively, both superior to those of the commercial reference gel. These results demonstrate the enhanced heat dissipation capability and promising application potential of the developed material in thermal management systems.

Through comprehensive evaluations of curing rheological properties and long-term reliability, the self-developed thermally conductive gel exhibits significantly higher and more stable bonding strength and thermal conductivity compared to the commercial counterpart. These findings provide robust experimental evidence supporting the application of the self-developed gel as a thermal interface material for TIM-I applications.

## 4. Materials and Methods

**Figure 10:**
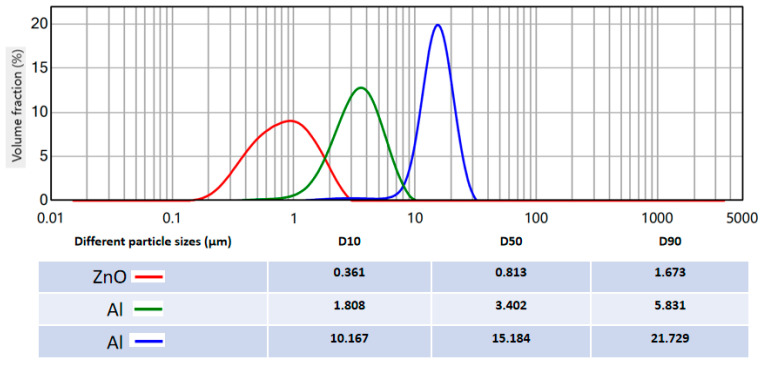
The particle size distribution profiles of the powders.

In this study, the volume-based particle size distribution (PSD) of zinc oxide (ZnO) and two aluminum (Al) powders with different particle size grades was determined via laser diffraction analysis, as illustrated in Figure 10. All three samples presented typical unimodal PSD curves with narrow distribution widths, indicating high particle size homogeneity. The ZnO powder exhibited the finest particle size, with a distribution peak centered at approximately 0.8 μm, while the green and blue Al powder samples showed progressively coarser particle sizes, with distribution peaks at ~3.4 μm and ~15.2 μm, respectively.

The key characteristic particle size parameters (D10, D50, D90) calculated from the PSD curves are listed in Figure 10. For the ZnO sample, the D10, D50, and D90 values were 0.361 μm, 0.813 μm, and 1.673 μm, respectively, confirming its ultrafine particle size. The Al powder with a medium particle size (green curve) had D10, D50, and D90 values of 1.808 μm, 3.402 μm, and 5.831 μm, respectively, while the coarse Al powder (blue curve) showed significantly larger values of 10.167 μm, 15.184 μm, and 21.729 μm for D10, D50, and D90, respectively. The span values (calculated as (D90-D10)/D50) for ZnO, medium Al, and coarse Al were 1.615, 1.182, and 0.761, respectively, further verifying the excellent particle size uniformity of all three samples.

As analyzed above, such a particle size distribution was tailored to achieve maximum thermal conductivity [45]. The above mixed filler, composed of Al powder and ZnO (model AZ300, Dongguan Eunow Chemical Co., Ltd., Dongguan, China), was employed as the thermally conductive filler. Vinyl silicone oil (model VS100, Jiangmen U-Bond New Material Co., Ltd., Jiangmen, China) was used as the gel matrix, while hydrogen-containing silicone oil (model HS200, Jiangmen U-Bond New Material Co., Ltd., China) served as the crosslinking agent. Alkynol (model BY400, Dongguan Eunow Chemical Co., Ltd., Dongguan, China) was used as the inhibitor, and a platinum complex catalyst (model PT500, Jiangmen U-Bond New Material Co., Ltd., Jiangmen, China) was adopted as the curing catalyst.

**Figure 11:**
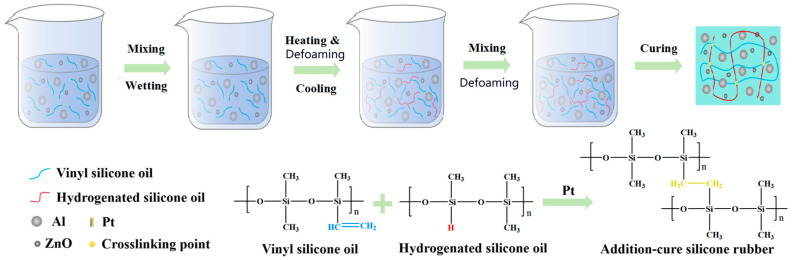
Flow chart of the thermal conductive silicone gel preparation.

**Figure 12:**
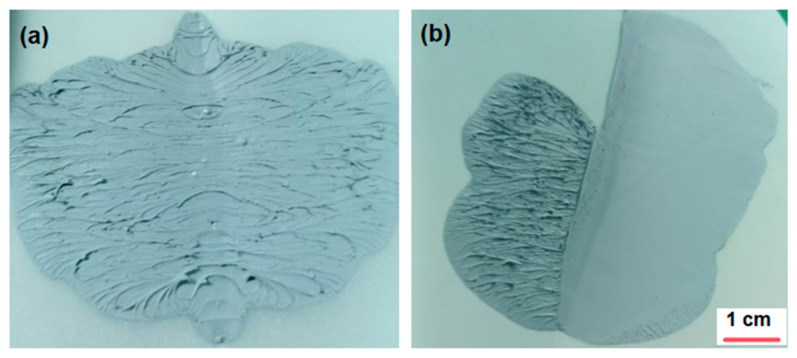
Visual comparison of the samples before (**a**) and after (**b**) achieving uniform dispersion.

The preparation procedure of the gel is illustrated in Figure 11. Vinyl silicone oil (5–15 wt%), hydrogen-containing silicone oil (1.8 wt%), mixed filler (aluminum powder and zinc oxide, 87–89 wt%), and platinum complex (0.2 wt%) were weighed according to the designed formulation. A DLH-5L power mixer (Foshan Jinyinhe Intelligent Equipment Co., Ltd., Foshan, China) was employed for the preparation process. Initially, vinyl silicone oil and the mixed filler were blended and stirred at room temperature for 120–180 min to ensure uniform dispersion, followed by standing for 14–18 h. The mixer features a dual-planetary agitation structure. The impellers execute simultaneous revolution and rotation, generating intense shear forces and kneading effects. This mechanism effectively disaggregates agglomerated powders, ensuring rapid dispersion of the adhesive within a short time frame. The agitation speed was 30–50 revolutions per min at room temperature for 120–180 min to ensure uniform dispersion. The dispersion uniformity of the mixture was evaluated as illustrated in Figure 12. A thin layer of the mixture was applied onto a release paper, folded in half along the coated surface, and then unfolded for visual inspection. Upon preliminary uniformity, the sample was further smoothed with a scraper to examine the internal homogeneity.

As depicted in Figure 12a, mixtures stirred for less than 120 min contained residual particles and were thus regarded as not having achieved uniform dispersion. In contrast, mixtures stirred for 120–180 min exhibited no visible particles either on the folded interface or after scraping, indicating satisfactory dispersion uniformity.

The dispersion was deemed uniform when the sample exhibited a particle-free morphology without chromatic aberration or heterochromatic spots. After confirming homogeneous dispersion, the mixture was heated to 130–180 °C under vacuum to remove residual moisture. Subsequently, the system was cooled to room temperature, and hydrogen-containing silicone oil, together with alkynol, was added. The mixture was further dispersed uniformly and subjected to vacuum treatment. Finally, the platinum complex catalyst was incorporated, and the conductive gel was obtained after thorough dispersion and vacuum defoaming.

According to recent market research conducted by Data Insights Market [46], the TIM-I segment exhibits a relatively high market concentration, with major players including Shin-Etsu, Dow, and Henkel dominating the field. Among the key suppliers, Shin-Etsu Chemical Co., Ltd. is considered a leading manufacturer. As a result, to evaluate performance at both the material and device levels, a commercial thermal conductive gel (X23-7772-4, Shin-Etsu Chemical Co., Ltd., Tokyo, Japan) was employed as the control sample, which was also composed of Al powder, ZnO powder, vinyl silicone oil, and hydrogen-containing silicone oil. For clarity, the self-developed thermal conductive gel and the commercial product are hereafter denoted as thermal conductive gel A and thermal conductive gel B, respectively.

Characterization method: Rheological properties were characterized using an Anton Paar MCR 302e rheometer (Anton Paar GmbH, Graz, Austria). The viscosity was measured using a variable shear rate program. A disposable PP25 parallel plate geometry with a gap of 0.6 mm was used. The measurement was conducted at a constant temperature of 25 °C, and the viscosity value at a shear rate of 10 s^−1^ was recorded. The gel time was determined using an oscillatory temperature ramp (T-Ramp) program. A disposable PP25 parallel plate geometry with a gap of 0.6 mm was employed. The measurements were performed at a constant strain of 10% and an angular frequency of 1 rad·s^−1^. The temperature program consisted of a ramp from 25 °C to 125 °C at a heating rate of 5 °C·min^−1^, followed by an isothermal hold at 125 °C for 30 min. The bonding strength of the thermally conductive gels under different processing parameters was measured using a tensile shear tester (DAGE 4000, Nordson Corp., Buckinghamshire, UK). The gel was dispensed onto a 25.4 × 25.4 mm copper substrate with nickel plating using an automatic dispensing machine, following a star-shaped (cross) path pattern. Subsequently, a 10 × 10 mm Si chip was pressed onto the gel with a pressure of 5 N. After thermal curing, the specimens for adhesion strength testing were obtained. The bonding area of the Si chip and the copper substrate with nickel plating was 100 mm^2^. The shear test was carried out on the DAGE tester in a horizontal pushing mode at a speed of 100 mm/min. According to the ASTM D5470-17 standard [47], the thermal conductivity was determined using a steady-state heat flow apparatus (LW-9389, Ruiling Instruments, Taiwan, China). Based on the IEC 61189-2-808 international standard, the contact thermal resistance under service conditions was evaluated using a thermal transient tester (Simcenter T3STER SI, Siemens EDA, Budapest, Hungary). The minimum bond-line thickness (BLT) and the filler morphology of the thermal conductive gels were characterized using an ultra-depth-of-field microscope (DVM6, Leica Microsystems, Wetzlar, Germany) and a scanning electron microscope (Apreo 2, Thermo Fisher Scientific Inc., Waltham, MA 02451, USA), respectively.

The long-term reliability evaluation was conducted under the following conditions: HTST was conducted in accordance with the JEDEC JESD22-A103 standard [48] under the condition of 150 °C for 1000 h. TCT was performed following the JEDEC JESD22-A104 standard [49], with a temperature range of −40 to 125 °C for 1000 cycles. uHAST per JEDEC JESD22-A118 standard [50] was conducted under high pressure, at 130 °C, and 85% relative humidity, without electrical bias.

## Figures and Tables

**Table 1:** Orthogonal experimental design of filler loading, silicone oil viscosity, and wetting time.

Serial Number	Filler Ratio (%)	Silicone Oil Viscosity (cP)	Wetting Time (h)
1	88	600	14
2	88	645	18
3	88	568	10
4	89	600	18
5	89	645	10
6	89	568	14
7	87	600	10
8	87	645	14
9	87	568	18

**Table 2:** Temperature of the cold end and hot end of the steady-state heat flow device under different compressed thicknesses.

Gel Type	Heat Transfer Area (mm)	Compression Thickness (mm)	Hot End Temperature (°C)	Cold End Temperature (°C)	Input Power (W)	Thermal Resistance (°C·W^−1^)
Thermal conductive gel A	6.452	0.337	50	45.45	28.46	0.157 ± 0.004
	6.452	0.221	50	46.59	30.31	0.105 ± 0.005
	6.452	0.107	50	47.89	33.82	0.062 ± 0.003
Thermal conductive gel B	6.452	0.337	50	45.82	28.61	0.159 ± 0.005
	6.452	0.221	50	47.10	31.22	0.109 ± 0.003
	6.452	0.107	50	48.21	34.36	0.057 ± 0.004

## Data Availability

The data supporting the findings of this study are available from the corresponding author upon reasonable request.
